# The role of mucosal-associated invariant T cells in visceral leishmaniasis

**DOI:** 10.3389/fimmu.2022.926446

**Published:** 2022-09-15

**Authors:** Marcela de Lima Moreira, Luana Oliveira Borges-Fernandes, Marcelo Antônio Pascoal-Xavier, Ágata Lopes Ribeiro, Victória Hellena Silva Pereira, Troi Pediongco, Márcio Sobreira da Silva Araújo, Andréa Teixeira-Carvalho, Andrea Lucchesi de Carvalho, Maria Vitória Assumpção Mourão, Flávia Alves Campos, Marineide Borges, Mariângela Carneiro, Zhenjun Chen, Eleanor Saunders, Malcolm McConville, Moriya Tsuji, James McCluskey, Olindo Assis Martins-Filho, Sidonia Barbara Guiomar Eckle, Jordana Grazziela Alves Coelho-dos-Reis, Vanessa Peruhype-Magalhães

**Affiliations:** ^1^ René Rachou Institute, Oswaldo Cruz Foundation (FIOCRUZ-MINAS), Belo Horizonte, Minas Gerais, Brazil; ^2^ Department of Microbiology and Immunology, The University of Melbourne at the Peter Doherty Institute for Infection and Immunity, Melbourne, VIC, Australia; ^3^ School of Medicine, Federal University of Minas Gerais, Belo Horizonte, Minas Gerais, Brazil; ^4^ João Paulo II Children’s Hospital, Fundação Hospitalar do Estado de Minas Gerais, Belo Horizonte, Minas Gerais, Brazil; ^5^ Parasitology Department, Universidade Federal de Minas Gerais, Belo Horizonte, Minas Gerais, Brazil; ^6^ Department of Biochemistry and Molecular Biology, The University of Melbourne, Melbourne, VIC, Australia; ^7^ Aaron Diamond AIDS Research Center, Department of Medicine, Columbia University Irving Medical Center, New York, NY, United States; ^8^ Department of Microbiology, Institute for Biological Sciences, Federal University of Minas Gerais, Belo Horizonte, Minas Gerais, Brazil

**Keywords:** MAIT, *Leishmania*, anti-parasitic activity, MR1, IL-17^+^MAIT, IFN-γ^+^ MAIT, TNF^+^MAIT

## Abstract

Mucosal-associated invariant T (MAIT) cells are restricted by MR1 and are known to protect against bacterial and viral infections. Our understanding of the role of MAIT cells in parasitic infections, such as visceral leishmaniasis (VL) caused by protozoan parasites of *Leishmania donovani*, is limited. This study showed that in response to *L. infantum*, human peripheral blood MAIT cells from children with leishmaniasis produced TNF and IFN-γ in an MR1-dependent manner. The overall frequency of MAIT cells was inversely correlated with alanine aminotransferase levels, a specific marker of liver damage strongly associated with severe hepatic involvement in VL. In addition, there was a positive correlation between total protein levels and the frequency of IL-17A^+^ CD8^+^ MAIT cells, whereby reduced total protein levels are a marker of liver and kidney damage. Furthermore, the frequencies of IFN-γ^+^ and IL-10^+^ MAIT cells were inversely correlated with hemoglobin levels, a marker of severe anemia. In asymptomatic individuals and VL patients after treatment, MAIT cells also produced IL-17A, a cytokine signature associated with resistance to visceral leishmaniasis, suggesting that MAIT cells play important role in protecting against VL. In summary, these results broaden our understanding of MAIT-cell immunity to include protection against parasitic infections, with implications for MAIT-cell-based therapeutics and vaccines. At last, this study paves the way for the investigation of putative MAIT cell antigens that could exist in the context of *Leishmania* infection.

## Introduction

Mucosal-associated invariant T (MAIT) cells are a subset of unconventional T-cells that expresses an invariant TCR α-chain, composed of the variable region 1-2 and joining region 33 (TRAV1-2-TRAJ33 in humans and TRAV1-TRAJ33 in mice), while TRAJ12 and TRAJ20 are also commonly incorporated in humans. Human MAIT cells can be identified by their expression of the α-chain along with a high expression of CD161 (1–8). MAIT cells are present in most tissues (9), are abundant in the human peripheral blood, where they represent on average 3% of T-cells (10) and are the most dominant T-cell subset in the human liver, where they comprise 20-50% of all T-cells (2, 11). MAIT cells are stimulated by a modified intermediate of bacterial/fungal vitamin B2 biosynthesis presented by the MHC-I-like MHC-I related protein 1 (MR1) (12–15). Upon activation, MAIT cells rapidly secrete T helper (Th)1 and Th17 cytokines (10, 16, 17) and cytotoxic granules (18) and provide first-line surveillance against lung bacteria (19–24). Despite advances in our understanding of MAIT cell biology, little is known about the role of these cells in protozoan infectious diseases.

*Leishmania* is a protozoan parasite that infects vertebrates, including humans, and causes leishmaniasis. Two strains of *Leishmania*, *L. infantum* and *L. donovani*, can cause the most severe form of leishmaniasis, i.e., visceral leishmaniasis (VL), which affects tropical areas such as Africa, India, and Brazil (25, 26). Although 80-90% of *L. infantum/donovani-*infected individuals remain asymptomatic, a severe form of the disease can occur, in which patients may develop severe anemia and leukopenia, persistent fever, hepatosplenomegaly, hemorrhagic manifestations and, ultimately, sepsis (27, 28). This aggravated state is characterized by a failure to generate an immediate cellular immune response against the pathogen, leading to the accumulation of malfunctioning T-cells and an exaggerated inflammatory cytokine response (29, 30). Overall, the role of conventional T-cells in visceral leishmaniasis has been described as either dysfunctional or deleterious to disease outcome, and restoration of T-cell responses is important for human recovery and resistance to reinfection in mice (31–35).

Here, we describe MR1-dependent activation of MAIT cells in the context of *Leishmania* infection and shed light on the putative role of MAIT cells in possibly limiting pathology in visceral leishmaniasis. To our knowledge, this is the first report of MR1-restricted responses induced by leishmania parasites.

## Results

### Circulating MAIT cells in children with active leishmaniasis are conspicuously decreased

**Figure 1:**
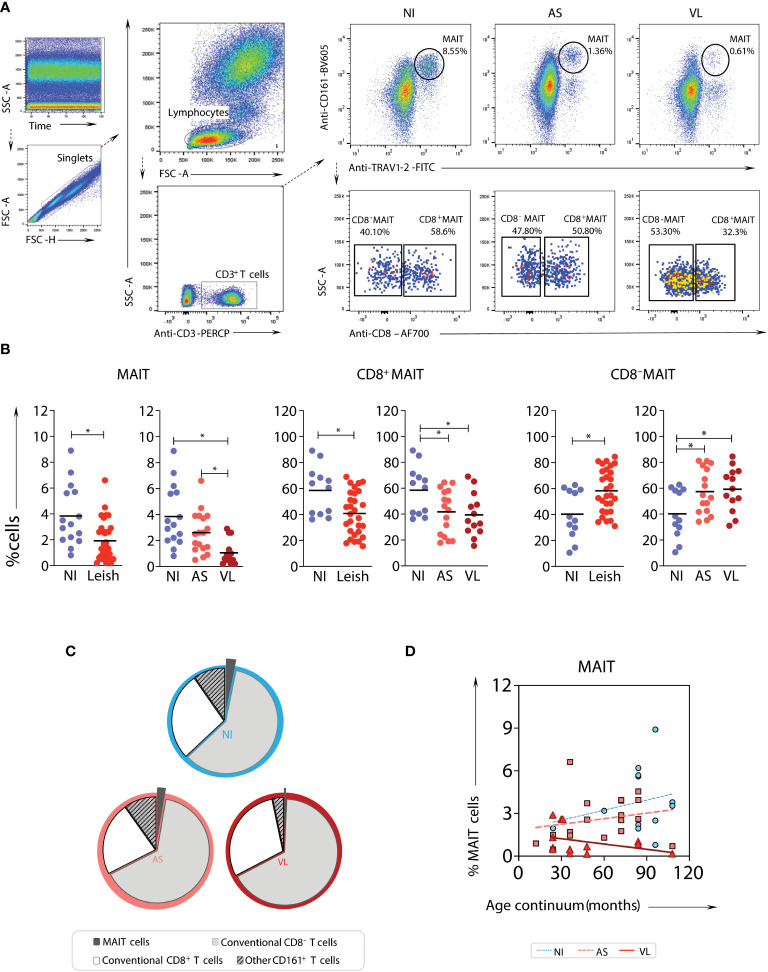
*Frequency and profile of MAIT cell subsets in samples from children with visceral leishmaniasis.* Multicolor flow cytometric analyses of TRAV1-2^+^CD161^high^ T-cells in peripheral whole blood samples of children with visceral leishmaniasis (VL = 14), asymptomatic carriers (AS = 18), and endemic non-infected controls (NI = 15). **(A)** Representative flow cytometric pseudocolor plots of NI, AS, and VL samples showing the gating strategy to identify TRAV1-2^+^CD161^high^ T-cells (referred to here as MAIT cells) and contour plots of CD8^+^ versus CD8^-^ TRAV1-2^+^CD161^high^ T-cells. **(B)** Scatter plot distribution of individual values with the median lines of the frequencies (%) of total CD3^+^ cells – MAIT cells (TRAV1-2^+^CD161^high^), and CD8α^-^, and CD8α^+^ MAIT-cell subsets in NI, Leish (AS+VL), AS, and VL samples. Significant differences amongst groups (p < 0.05) based on the Mann-Whitney test for comparing two groups are indicated by connecting lines and asterisks (*p ≤ 0.05). **(C)** Pie charts displaying the mean proportions of MAIT cells (black), non-MAIT (TRAV1^-^2^-^ CD161^low/-^) CD8^-^ T-cells (light grey), non-MAIT CD8^+^ T-cells (dark grey), and other CD161^+^ T-cells (mid-grey) in peripheral blood amongst VL, AS, and NI samples. **(D)** Linear regression analyses for MAIT cell frequency according to the age group for VL (continuous line), AS (dashed line), and NI (dotted line), assessing the impact of age on the percentage of circulating TRAV1-2^+^ CD161^high^ T-cell subsets.

Peripheral blood samples from 2 to 10 years old children with visceral leishmaniasis (Leish), comprised of asymptomatic (AS) and active (VL) cases as well as non-infected endemic healthy controls (NI), were evaluated. The frequencies of total MAIT cells and CD8^-^ and CD8^+^ MAIT cell subsets were assessed (**Figures 1A, B**). In the absence of tetramers, MAIT cells were identified based on surrogate markers (CD161^high^TRAV1-2^+^) (**Figure 1A**). A significant decrease in total MAIT cells in the peripheral blood of Leish patients - AS and VL subgroups - was observed compared to NI. In the VL subgroup, there was a decrease in the fraction of MAIT cells compared to the AS and NI subgroups (**Figures 1B, C**). Notably, the frequency of the CD8^+^ MAIT cell subset was reduced in VL compared to NI (**Figure 1B**). To determine whether age impacted the percentage of circulating MAIT cells and subsets in VL children, comparative linear regression analyses were conducted for each MAIT cell subset frequency according to age group. This analysis revealed that older children presented the most significant decrease in MAIT cell frequencies when comparing VL with AS and NI (**Figure 1D**).

### MAIT cells from asymptomatic children display a prominent proinflammatory profile after *in vitro* exposure to live *L. infantum*

**Figure 2:**
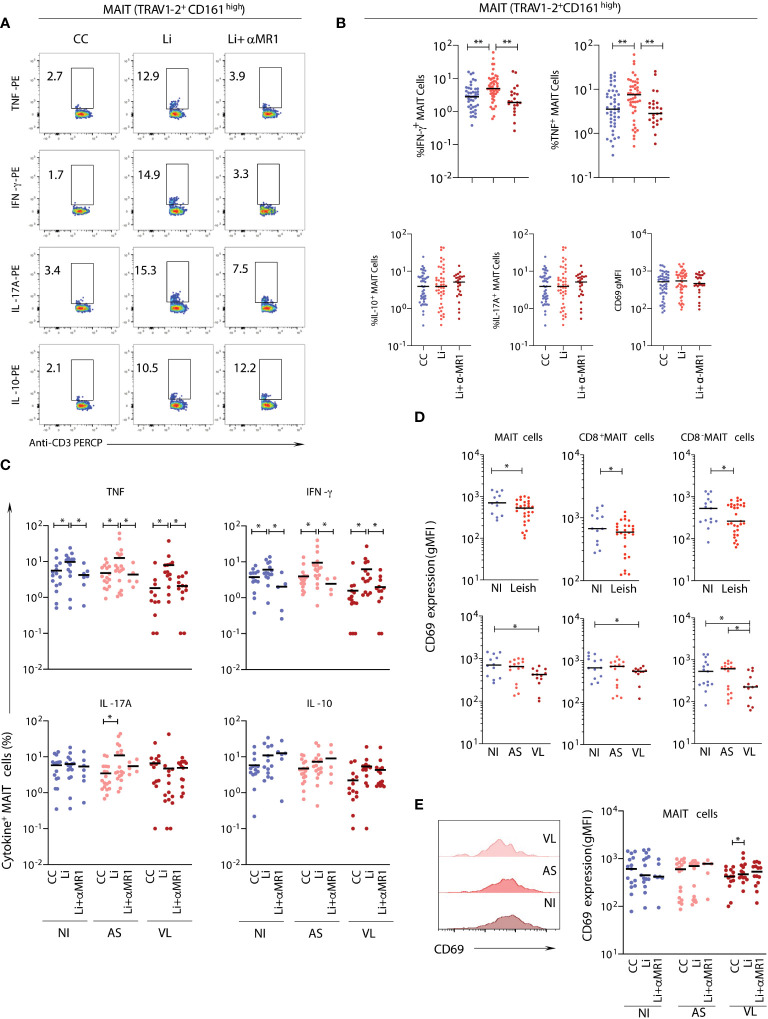
*In vitro stimulation of MAIT cells from samples of children with asymptomatic and active visceral leishmaniasis*. Plasma-depleted peripheral whole-blood samples of children with visceral leishmaniasis (VL = 14), asymptomatic carriers (AS = 18), and endemic non-infected controls (NI = 15), as in **Figure 1**, were short-term incubated with promastigotes with (Li+αMR1) or without (Li) MR1 blocking antibody and the expression of intracellular cytokine, as well as a surface activation marker, were evaluated. Significant differences amongst groups (p<0.05) based on the Mann-Whitney test for comparing two groups are indicated by connecting lines and asterisks (*p ≤ 0.05; **p ≤ 0.01). **(A)** Representative flow cytometry pseudocolor dot plots (large dots) showing gating of cytokine^+^ MAIT cells after *in vitro* culture – control (CC), Li and Li + αMR1. **(B)** Scattering distribution of individual values with the median lines showing the proportion of MAIT cells expressing TNF, IFN-γ, IL-17A, and IL-10 and CD69 expression (geometric mean fluorescence intensity-gMFI) as a marker of activation in all samples pooled together (NI, AS and VL) in CC, Li, and Li+αMR1 cultures. **(C)** Scattering distribution of individual values with the median lines showing the proportion of MAIT cells expressing TNF, IFN-γ, IL-17A and IL-10 in NI, AS, and VL samples for CC, Li and Li + αMR1 cultures. The number samples included in the Li + αMR1 condition for asymptomatic carriers and endemic non-infected controls groups was AS = 5 and NI = 7. **(D)** Scattering distribution of individual values with the median lines of CD69 expression (gMFI) *ex vivo* by MAIT cells, CD8α^-^ and CD8α^+^ MAIT cells in NI, Leish, AS, and VL samples for control cultures (CC). **(E)** Representative flow cytometry histograms of CD69 expression in NI, AS, and VL samples, and box and whiskers plot with quartiles, median, and min to max values of the CD69 expression (gMFI) by MAIT cells in NI, AS, and VL samples after *in vitro* cultures CC, Li, and Li+αMR1.

To evaluate the impact of live *L. infantum* exposure on MAIT-cell function, including MR1 dependency, peripheral blood samples from NI and Leish (AS and VL) patients were stimulated with live *L. infantum* promastigotes in the presence or absence of anti-MR1 antibody, and the frequencies of proinflammatory (TNF, IFN-γ as well as IL-17A) and regulatory (IL-10) cytokine-producing MAIT cells were measured (**Figure 2A**). Overall, for all patient samples, similar percentages of MAIT cells produced TNF and IFN-γ upon *in vitro L. infantum* exposure, and cytokine production was abrogated by MR1 blockade (**Figures 2A, B**). MAIT cells also produced TNF and IFN-γ in an MR1-dependent manner upon *in vitro L. infantum* stimuli when NI, AS, and VL subgroups were evaluated individually (**Figure 2C**). In AS children, but not in VL or NI, MR1-independent IL-17A production was observed after *L. infantum* stimulation (**Figure 2C**). Therefore, NI, AS, and VL displayed a Type1 response, which was the major MR1-dependent response, while a Type17 MR1-independent response was observed exclusively in AS children (**Figure 2C**).

MAIT cells, including the CD8^-^ and CD8^+^ subsets, showed a decrease in CD69 expression levels in the VL group compared to the NI group, which was not observed in the AS group (**Figures 2D, E**). Following live *L. infantum* exposure, no differences in CD69 levels were observed in NI and AS groups, but an MR1-dependent upregulation of CD69 was found in the VL group (**Figures 2D, E**).

### Aspects of the MAIT cell profile in visceral leishmaniasis patients are associated with biochemical and clinical findings related to disease severity

**Figure 3:**
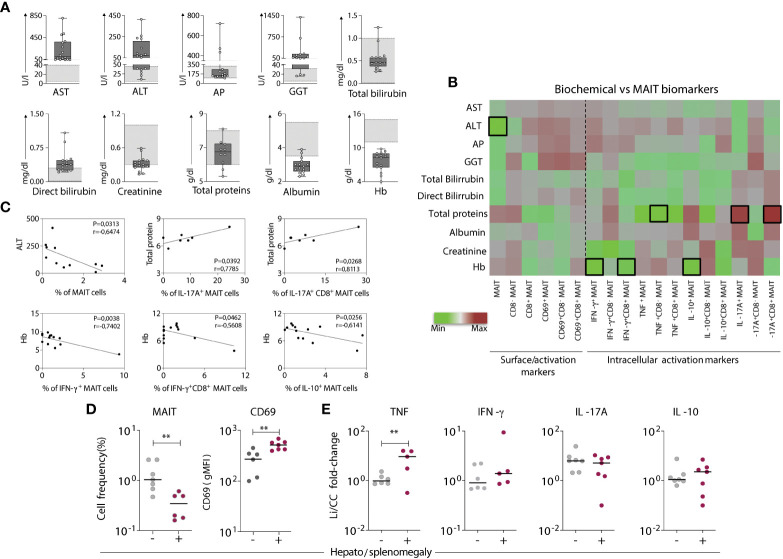
Aspects of the MAIT cell profile are associated with biochemical and clinical findings related to the severity of visceral leishmaniasis. **(A)** Scattering distribution of individual values over box plots underscoring the median values, interquartile ranges along with min to max values of each biochemical liver biomarker (evaluated using standard clinical testing) in VL patients compared to reference values (light grey bars). Biochemical markers include aspartate transaminase-AST, alanine transaminase-ALT, alkaline phosphatase-AP, gamma-glutamyl transferase-GGT, total and direct bilirubin, total protein, albumin, creatinine, and hemoglobin-Hb. **(B)** Heatmap chart displaying the correlation between the phenotype/function of various MAIT cell subsets obtained by fold-changes of Leishmania-infected cultures and the respective control culture (Li/CC) and the biochemical liver biomarkers evaluated for each VL patient. Colors express the intensity of correlation (-1 < R < 1) as defined by the indicated color key. Black bordered squares highlight statistically significant correlations (p < 0.05). **(C)** Linear regression analysis of the biochemical liver biomarkers according to MAIT cell biomarkers. **(D)** Scattering distribution of individual values with the median lines of MAIT cell frequency amongst T-cells and CD69 expression (gMFI) in VL patients grouped based on the absence **(-)** and presence (+) of hepatosplenomegaly. **(E)** Fold-changes in cytokine expressing MAIT cells. Fold changes were calculated using results of *Leishmania*-infected cultures and the respective control culture (Li/CC) from VL patients grouped based on the absence **(-)** and presence (+) of hepatosplenomegaly. Significant differences amongst groups (p<0.05) based on Mann-Whitney test are indicated by connecting lines and asterisks (**p ≤ 0.01).

To understand whether the functional properties of MAIT cells in the VL group were associated with disease pathology, relevant biochemical and clinical parameters (aspartate transaminase-AST, alanine transaminase-ALT, alkaline phosphatase-AP, gamma-glutamyl transferase-GGT, total and direct bilirubin, total protein, albumin, creatinine, and hemoglobin-Hb) were evaluated (**Table 1** and **Figure 3A**). Then, these biochemical and clinical parameters were correlated with the overall MAIT cell frequency and frequencies of various MAIT cell subsets, such as cytokine-producing MAIT cells. For this analysis, fold-changes in *Leishmania*-infected cultures according to the respective control culture were calculated and correlated with a range of biochemical parameters. The overall frequency of MAIT cells was negatively correlated with ALT, a classical laboratory marker of liver damage, strongly associated with severe hepatic involvement in VL (28, 36). In addition, there was a positive correlation between the total protein levels and the frequency of IL-17A^+^ CD8^+^ MAIT cells. The frequencies of IFN-γ^+^ and IL-10^+^ MAIT cells were inversely correlated with Hb levels, a marker of severe anemia (**Figures 3B, C**). Furthermore, VL patients presenting hepatosplenomegaly had severely reduced MAIT cell frequency and increased CD69 expression (**Figure 3D**). In *Leishmania*-infected cultures, VL patients had increased fold-change of TNF^+^ MAIT-cells compared to patients without those symptoms (**Figure 3E**). At the same time, no significant differences in the fold-change of IFN-γ^+^, IL-17A^+^ and IL-10^+^ MAIT cells were detected (**Figure 3E**). Overall, these findings reinforce the participation of MAIT cells in *Leishmania*-induced immunity during active disease, whereby MAIT cells appear to exert dual functions, i.e., protective and/or pathological inflammatory responses in VL patients.

**Table 1:** Demographic, clinical and laboratorial information of healthy endemic controls (NI) and Leishmaniasis patients classified as asymptomatic carriers (AS) and visceral leishmaniasis (VL).

Demographic and clinical/laboratorial data		Leish	
	NI	AS	VL
**N**	15	18	14
**Age (median)**	2-9 (7)	1-9 (5.5)	2-9 (2.5)
**Female (%)**	39	67	43
**Male (%)**	61	33	57
**Signs and symptoms**	NA	NA	Fever (86%), increased abdominal volume (43%), pancytopenia (29%)
**qPCR (%)**	0	17	-
**rK39 ELISA (%)**	0	100	–
**qPCR/rK39 ELISA (%)**	0	17	-
**IT-Leish (%)**	–	–	100
**AST (median) U/L**	NA	NA	42-804 (149.50)
**GPT (median) U/L**	NA	NA	11-415 (78.50)
**ALT (median) U/L**	NA	NA	116-721 (165.00)
**GGT (median) U/L**	NA	NA	16->1400 (80.00)
**TB (median) mg/dL**	NA	NA	0.26-1.24 (0.45)
**DB (median) mg/dL**	NA	NA	0.21-1.08 (0.38)
**Total Proteins (median) g/L**	NA	NA	5.70-8.10 (6.75)
**Albumin (median) g/L**	NA	NA	2.30-3.90 (2.90)
**Urea (median) mg/dL**	NA	NA	12-25 (18.50)
**Creatinine (median) mg/dL**	NA	NA	0.13-0.59 (0.29)
**Hb (median) g/L**	NA	NA	3.80-9.80 (8.30)
**Meglumine Antimoniate (%)**	NA	NA	64
**Liposomal Amphotericin B (%)**	NA	NA	36

aspartate transaminase-AST, glutamic pyruvic transaminase (GPT), alanine transaminase-ALT, gamma glutamyl transferase-GGT, total-TB and direct bilirubin-DB, total protein, albumin, urea, creatinine and hemoglobin-Hb.

### Response of CD8^-^ and CD8^+^ T-cells after *in vitro L. infantum* stimuli

**Figure 4:**
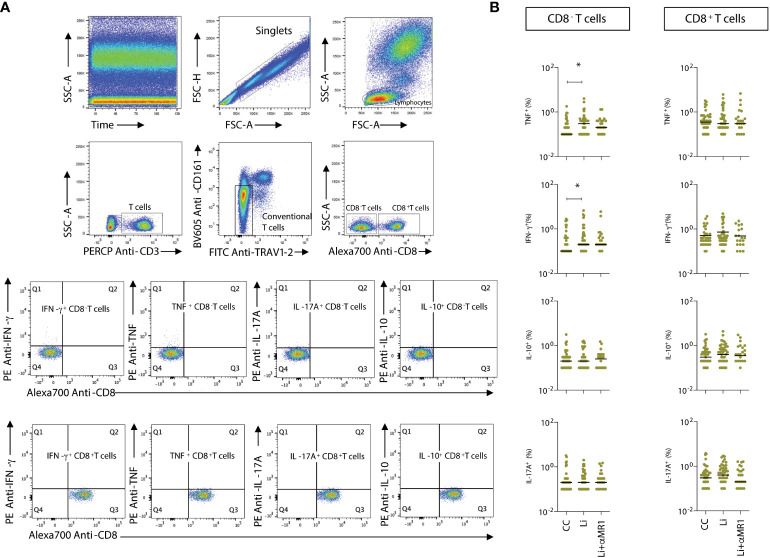
L. infantum*-induced production of proinflammatory cytokines by non-MAIT T-cells.* **(A)** Representative flow cytometry pseudocolor plots showing gating of cytokine^+^ CD8α^-^ and CD8α^+^ non- MAIT cells after *in vitro* culture – CC, Li, and Li+αMR1. **(B)** Scattering distribution of individual values with the median lines of frequencies (%) of intracellular cytokine-producing non-MAIT CD8^-^ and CD8^+^ T-cells in all samples (NI, AS and VL), as part of the same experiment displayed in **Figure 2B**. Significant differences amongst groups (p<0.05) based on the Mann-Whitney test for comparing two groups are indicated by connecting lines and asterisks (*p ≤ 0.05).

To determine the potential impact of MR1-dependent MAIT cell activation on non-MAIT T-cells, intracellular cytokine (TNF, IFN-γ, IL-17A, and IL-10) production by non-MAIT TRAV1-2^-^ CD161^low/-^ CD8^-^ and CD8^+^ T-cells was evaluated upon stimulation with *L. infantum* in the absence or presence of MR1 blockade in all children (**Figure 4A**). Small proportions of CD8^-^ and CD8^+^ non-MAIT T-cells in all children produced TNF and IFN-γ after *L. infantum* stimuli, which were not affected by MR1 blockade (**Figure 4B**). Pseudo-heatmap analysis (**Supplementary Figure 1**) indicated that while there was an increased number of subjects with higher proportions of proinflammatory cytokine-producing CD8^-^ and CD8^+^ T-cells in AS after MR1 blocking, it correlated with fewer subjects with higher frequencies of TNF^+^ and IFN-γ^+^ MAIT-cells. Alternatively, these data may indicate that MAIT cells exert the role of the predominant proinflammatory T-cell subset upon *Leishmania* short-term stimuli *in vitro*.

### MR1-dependent abrogation of soluble TNF, IFN-γ, and IL-17A secretion after whole blood short-term *in vitro* culture with *L. infantum*

**Figure 5:**
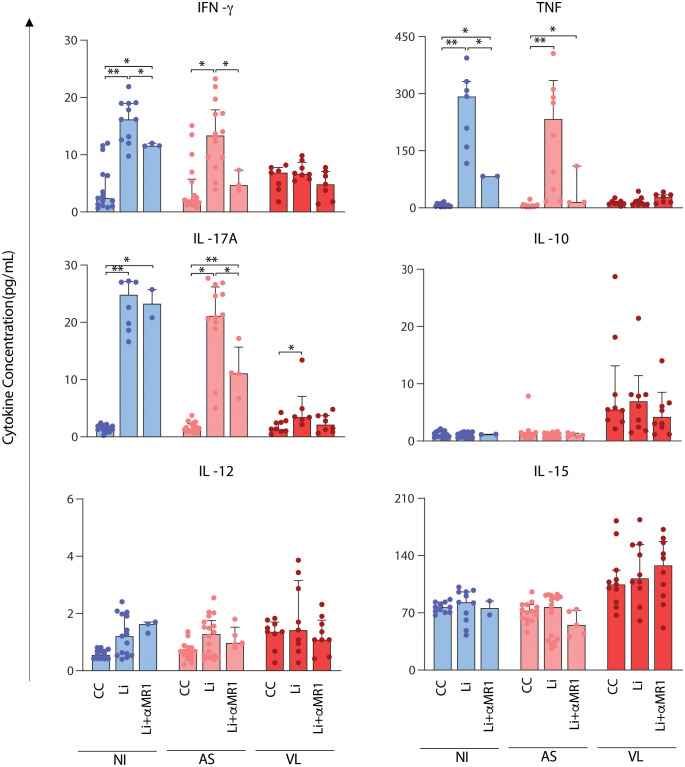
*MR1-dependent abrogation of soluble cytokine secretion after short-term in vitro culture with* L. infantum. Scattering distribution of individual values over bar charts with a median and interquartile range of TNF, IFN-γ, IL-17A, IL-10, IL-12, and IL-15 after *in vitro* culture – control (CC), Li, and Li + αMR1. Significant differences amongst groups (p < 0.05) based on the Mann-Whitney test for comparing two groups are indicated by connecting lines and asterisks (*p ≤ 0.05; **p ≤ 0.01).

Proinflammatory cytokines TNF, IFN-γ, IL-17A, IL-12, IL-15, and the regulatory cytokine IL-10 were quantified, and the results were expressed in pg/mL, as shown in **Figure 5**. The results demonstrate significant increase in IFN-γ, TNF, and IL-17A induced by *L. infantum* predominantly in NI and AS groups. In these groups, the increased IFN-γ and TNF production *in vitro* was abrogated by MR1 blockade, except for VL. For IL-17A, cytokine production was diminished in an MR1-dependent manner only in the AS group. No production of IL-10, IL-12, and IL-15 was observed after short-term culture with *L. infantum.* IL-10 and IL-15 were augmented consistently in VL compared to NI and AS, but with no alteration by *L. infantum* stimuli or MR1 blockade (**Figure 5**).

### Leishmanicidal drug treatment of VL patients affects the frequencies and functional profile of MAIT cells

**Figure 6:**
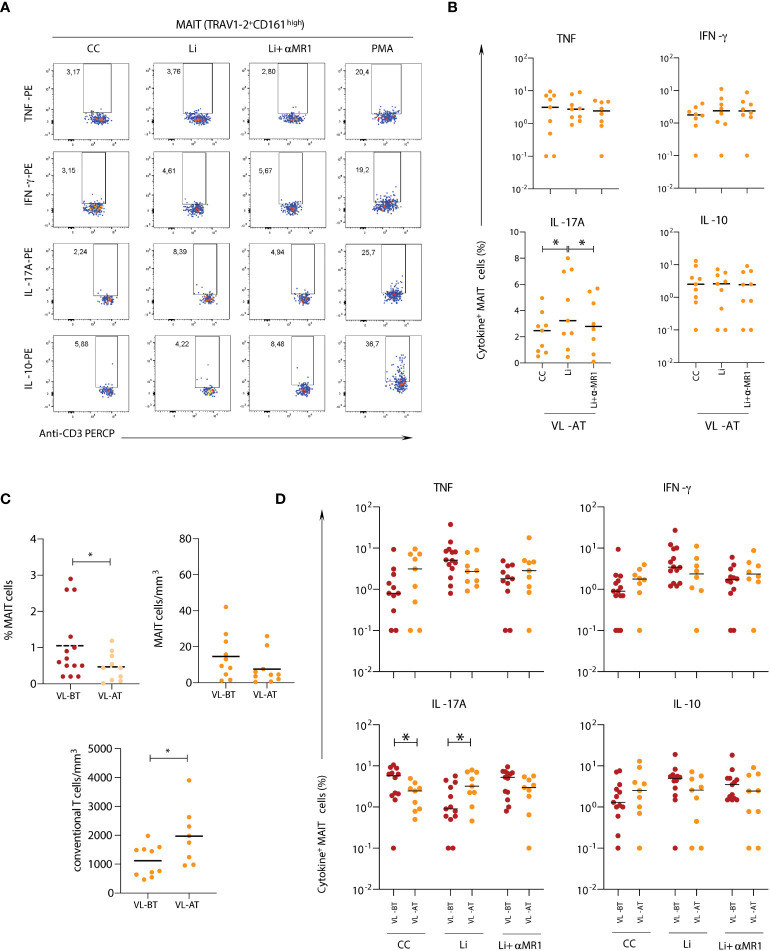
*Impact of leishmanicidal therapy in MAIT cell activation in response to* Leishmania. Multicolor flow cytometric analyses of TRAV1-2^+^CD161^high^ T-cells in peripheral whole blood samples of children with visceral leishmaniasis before (VL-BT) and after (VL-AT) treatment with Glucantime^®^ or Amphotericin B. **(A)** Representative flow cytometry pseudocolor dot plots showing gating of cytokine^+^ MAIT cells after *in vitro* culture in **(A)** it's Ctr in the panel, Li, and Li + αMR1. **(B)** Box and whisker plots with quartiles, median, and min to max values showing the proportion of MAIT cells expressing TNF, IFN-γ, IL-17A, and IL-10 in samples from VL-AT children in CC, Li, and Li + αMR1 cultures. **(C)** Scattering distribution of individual values with the median lines of the frequencies (%) for total CD3^+^ cells – MAIT cells (TRAV1-2^+^CD161^high^) and counts of MAIT cells and non-MAIT cells (TRAV1-2^-^CD161^-^), calculated based on lymphocyte differential counts from complete blood counts. **(D)** Scattering distribution of individual values with the median lines showing the proportion of MAIT cells expressing TNF, IFN-γ, IL-17A, and IL-10 in VL-BT and VL-AT in CC, Li and Li + αMR1 cultures. Significant differences amongst groups (p<0.05) based on the Mann-Whitney test for comparing two groups are indicated by connecting lines and asterisks (*p ≤ 0.05).

To understand if leishmanicidal drug treatment could affect MAIT cell frequencies and function in VL children, we evaluated the patients after 1 to 2 months of treatment with Glucantime^®^ or Amphotericin B (VL-AT) (**Figures 6A, B**). After *in vitro* stimulation with *Leishmania*, MAIT cells from VL-AT children produced exclusively IL-17A, suggesting that MAIT cells expressing IL-17A were selectively preserved after infection resolution (**Figure 6B**). Unlike IL-17A produced by MAIT cells in AS patients (**Figure 2C**), in VL patients upon drug treatment, IL-17A production was MR1-dependent. Additionally, MAIT cell frequencies were reduced after treatment, consistent with a tendency to decrease the absolute numbers of circulating MAIT cells (**Figure 6C**). In contrast, non-MAIT T-cells showed a rapid increase after treatment, mainly due to increased CD8α^-^ non-MAIT T-cells (**Figure 6C**). In addition to the reduction of MAIT cells in VL-AT samples, there was a decrease in the frequency of IL-17A expressing MAIT cells *ex vivo* (Control culture) compared to samples before treatment (VL-BT). However, after *in vitro* stimulation with *Leishmania*, VL-AT samples presented an increased ability to produce IL-17A in response to the infection than VL-BT samples, in line with the hypothesis that IL-17A^+^ MAIT cells are preferentially selected after drug treatment (**Figure 6D**).

### Leishmania *in vitro* infection induces a dose-dependent activation of a fraction of 5-OP-RU-MR1-restricted MAIT cells

**Figure 7:**
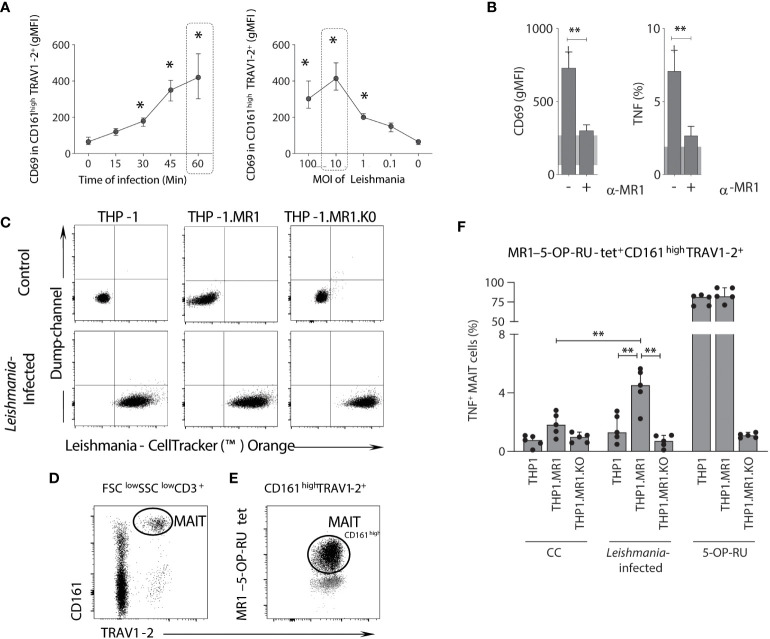
*MAIT cell activation following in vitro* Leishmania *infection is dependent on MR1, infection time and dose*. **(A)** Connecting line XY-graph showing the median with range (min and max) of surface-expressed CD69 (gMFI) on TRAV1-2^+^CD161^high^ (MAIT) cells after *in vitro* stimulation with *L. infantum*, according to the time of infection and MOI before 4h incubation with brefeldin. MAIT cells were analyzed using plots similar to the ones shown in **Figure 1**. **(B)** Bar graphs representing the median with range (min and max) of surface CD69 expression (gMFI) and percentage of intracellular TNF-producing MAIT cells in plasma depleted whole-blood samples (healthy donors, n = 5) following *in vitro L. infantum* stimulation using the conditions highlighted with dashed rectangles in panel A, in the presence or absence of anti-MR1 blocking antibody (clone 26.5). The shaded grey represents the 95% confidence interval from control culture. **(C)** Flow cytometric dot plots of control (non-infected) and *Leishmania*-infected THP-1, THP-1.MR1 and THP-1.MR1.K0 cells. **(D, E)** Representative flow cytometric dot plots showing the gating strategy to identify MAIT cells amongst CD3^+^ T cells in peripheral blood mononuclear cells from healthy donors after *in vitro* stimulation with *L. donovani* based on TRAV1-2^+^CD161^high^ **(D)** and 5-OP-RU-MR1 tetramer back-gated for TRAV1-2^+^ CD161^high^ and CD161^low^ expression before the selection of TNF-producing MAIT cells **(E)**. **(F)** Bar graph displaying median with range (min and max) of the frequency of intracellular TNF-producing MAIT cells in conditions as indicated, using *Leishmania donovani* for infection of THP-1 cell lines, co-incubated with PBMCs samples (5 healthy donors). Significant differences (p<0.05), based on the Mann-Whitney test for comparing two groups, are highlighted by connecting lines and asterisks (*p ≤ 0.05; **p ≤ 0.01).

To assess whether the activation of MAIT cells by *Leishmania* is triggered in a time-, dose-, and MR1-restricted manner, whole blood samples from healthy adults were incubated with live *Leishmania* promastigotes and the expression of CD69 by MAIT cells (CD161^high^TRAV1-2^+^ cells) was monitored from 0 to 60 minutes (before 4h incubation with brefeldin) and at 100 to 0.1 multiplicity of infection (MOI). Data demonstrated that CD69 upregulation was time- and dose-dependent, reaching higher levels at 60 minutes using an MOI of 10 (**Figure 7A**), and CD69 upregulation and TNF production were dependent on MR1 (**Figure 7B**).

To confirm the MR1 dependency during activation of MAIT cells by live *Leishmania* promastigotes, *L. donovani*, a previously developed panel of THP-1 monocytic cell lines (20), was employed as antigen-presenting cells (APCs), which included wild-type, MR1 overexpressing (THP-1.MR1) and MR1 knock-out (THP-1.MR1KO) cells. THP-1 cells were co-cultured in the presence of live *Leishmania* promastigotes stained with an orange cell tracker to demonstrate similar levels of *Leishmania* infection across the THP-1 cell lines (**Figure 7C**). MAIT cells, identified by MR1-5-OP-RU tetramer, were also co-stained with surrogate MAIT cell markers (CD161^high^ TRAV1-2^+^) (**Figure 7D**), suggesting that *Leishmania*-specific MR1-restricted MAIT cells are a subset of MR1-5-OP-RU specific T-cells (**Figure 7E**). After co-incubating *Leishmania*-infected THP-1 cells with peripheral blood mononuclear cells (PBMCs) from healthy adults, TNF production by MAIT cells (MR1-5-OP-RU tetramer^+^ cells) was dependent on MR1 with the highest frequency of TNF^+^ MAIT cells observed when co-incubated with *Leishmania* infected THP-1.MR1 cells as APCs (**Figure 7F**). Accordingly, MAIT cells responded to both *L. infantum* and *L. donovani* in an MR1-dependent manner.

## Discussion

MAIT cells have previously been shown to provide protective immunity against bacterial, fungal (16, 20, 22, 24, 37), and viral (38–40) infections. While their response to bacterial and fungal infections depends on the recognition of MR1 presentation of riboflavin pathway-derived antigens (14, 41), MAIT cell responses to viral agents are independent of MR1-Ag (39, 40). Here, we showed that MAIT cells also have a role in protecting from *Leishmania* parasites, thus broadening the spectrum of antimicrobial immune responses mediated by MAIT cells. Notably, the MAIT cell response against *Leishmania* was dose- and MR1-dependent. This response was characterized by TNF, IFN-γ, and IL-17A production, which was observed in intracellular staining and cytokines in culture supernatants. TNF, IFN-γ, and IL-17A have been strongly associated with protection during leishmaniasis (32, 33, 42–47). Given that *Leishmania* parasites do not code for riboflavin biosynthesis, this demonstrates the need for further study of new antigens in the context of *Leishmania* infection that might be similar between the two *Leishmania* species tested in this study (*L. infantum* and *L. donovani*). Cells responsive to parasite infection appeared to make up a fraction of MR1-5-OP-RU-restricted MAIT cells. The ability of the 5-OP-RU-specific MAIT TCR repertoire to cross-react with non-riboflavin pathway-derived antigens was previously observed for MAIT cells responding to small molecule drugs (48) and folate-based antigens (26). Whether there are also *Leishmania* responsive MR1-reactive T-cells outside the MR1-5-OP-RU-restricted repertoire remains to be investigated. Other MR1-reactive T-cells that do not respond to 5-OP-RU have previously been described (14, 15, 26). The variability observed for MAIT cell responses to *Leishmania* stimuli amongst individuals might imply differences in the repertoire of MAIT cells, potentially imposed by the microbiota or previous infections. The more pronounced response in AS compared to NI children to *in vitro Leishmania* infection suggests the pre-existence of larger numbers of *Leishmania* responsive clones.

Similar to previous studies on patients with acute or chronic diseases such as tuberculosis (6, 49, 50) and viral infections, including HIV, HCV, and Dengue virus (39, 40, 51), we also found a correlation between decreased frequencies of circulating MAIT cells and active disease in VL patients. Regarding the role of MAIT cells in active/chronic parasitic diseases, it was demonstrated that *Plasmodium falciparum* sporozoites may induce a reduction of circulating CD8^-/+^ MAIT cells (52). As speculated by previous studies, it is thus possible that also in active VL, MAIT cells migrate from the blood to the site of infection.

It is well established that active VL occurs following ineffective *L. donovani* or *L. infantum* control by infected cells, including macrophages, monocytes, and neutrophils (53–56). Active VL is characterized by a misbalance of TNF, IFN-γ, and regulatory IL-10, produced mainly by macrophages and regulatory T/B-cells (33, 42, 45, 57). In this study, the type-1 cytokine signature was strongly associated with MR1-dependent activation in the context of *Leishmania* infection *in vitro*. Although isotypic controls were not assessed for anti-MR1 blocking in the whole blood cultures of NI, AS, and VL due to children sampling restrictions, the results observed for the THP-1 cell lines corroborate the hypothesis of an MR1-dependent effect on MAIT cell response to *Leishmania*. While THP-1.MR1KO fails to induce cytokine production by MAIT cells, the over-expression of MR1 upregulates the TNF production by MAIT cells.

In addition, an exacerbated proinflammatory profile is associated with systemic inflammation in VL (29). Interestingly, MAIT cells from AS, but not NI or VL patients, also produced IL-17A in an MR1-dependent manner, which has been identified as beneficial in preventing the progression to VL (58).

In summary, distinct subsets of MAIT (Type1 and Type17) seem to have opposing roles during active *L. infantum* infection. MAIT cells did not produce intracellular or soluble IL-10 under live parasite stimulation. However, VL demonstrated consistently elevated levels of soluble IL-10 compared to NI and AS. IL-10 is the major suppressor of anti-*Leishmania* immune mechanisms in VL patients, promoting parasite persistence and susceptibility to severe disease (32, 33, 59–62). Other sources of IL-10, such as B-cells and macrophages, are possibly responsible for the total circulating IL-10 levels in VL children. In pre-exposed asymptomatic subjects, robust production of IFN-γ, TNF, and IL-17A indicates that in the absence of systemic inflammation, the two types of MAIT cell-mediated immune responses (Type1 and Type17) may synergize beneficially, counterbalanced by IL-10.

Regarding cytokines related to TCR-independent activation of MAIT cells, short-term culture with live parasite did not induce the production of IL-12 and IL-15, indicating that initial *L. infantum* activation of MAIT cells is strongly associated with a TCR-dependent activation pathway. These two cytokines may play a role in *L. infantum*-mediated MAIT cell activation in more extended incubation periods, which will be addressed in future studies.

In this study, no patient with active VL presented severe disease or mortality; yet our data suggested that MAIT cells contribute to this proinflammatory environment. In children with active VL, lower frequencies of circulating MAIT cells with increased levels of CD69 expression and higher proportions of TNF-producing cells were significantly associated with hepatosplenomegaly. The lower frequencies of MAIT cells also correlated with clinical markers of liver damage. It has been proposed that MAIT cells might contribute to acute liver injury (63). On the other hand, Jeffery *et al.* (64) proposed a protective role of MAIT cells in liver diseases associated with bacterial infection of different etiologies. Correlation analyses suggest that in active VL, the reduction of circulating MAIT cells can be related to an accumulation of this cell population in the liver and contribute to liver damage. More studies evaluating the profile of hepatic MAIT cells during human VL are needed to confirm this hypothesis. Moreover, the frequencies of circulating IFN-γ^+^ and IL-10^+^ MAIT cells were associated with a lower hemoglobin level, indicative of severe anemia. It has previously been shown that increased levels of circulating IL-10 in VL patients correlate with lower hemoglobin levels, which was directly associated with spleen and liver enlargement (29). However, we did not see a correlation between the frequency of IL-10-producing MAIT cells and liver/spleen enlargement. Thus, it is crucial to consider the subtle balance between immune response and clinical status in active VL.

Overall, our data expand the spectrum of the activity of MAIT cells by demonstrating its importance as the first line of defense against microbial and parasitic infections, specifically in asymptomatic visceral leishmaniasis. More studies are still needed to unveil the function of MAIT cells in the pivotal site of infection by parasites. These findings may shed light on the development of therapeutics and vaccines, an urgent necessity for controlling the spread of this fatal and highly neglected disease.

## Material and methods

### Clinical samples and ethics statements

VL-infected symptomatic children (VL group) residing in the metropolitan region of Belo Horizonte, Minas Gerais, Brazil, were recruited at the João Paulo II Children’s Hospital. VL children presented symptoms of active VL, positive serology for VL diagnosis was determined by IT Leish^®^ (Bio-Rad Laboratories, Hercules, CA, USA), and biochemical profiling was performed by conventional colorimetric methods at the hospital laboratory. Peripheral whole blood samples from 14 VL patients were collected before treatment. Asymptomatic children included in this study were positive in serological (rk39) or whole blood (buffy coat) real-time-PCR tests and lacked symptoms during clinical evaluation (within the last 3 years of follow-up before blood collection). Peripheral blood samples were collected from 18 asymptomatic children (AS group) and 15 non-infected (NI group) age and gender-matched healthy endemic controls recruited in the metropolitan region of Belo Horizonte. Demographic, clinical, and laboratory data are described in **Table 1**. All individuals included in this study agreed to participate and signed an informed written consent. This study followed the 466/2012 Brazilian resolution guidelines established by the National Commission on Research Ethics (CONEP) and was approved by the Ethics Committee at the René Rachou Institute (IRR/FIOCRUZ-MINAS protocol #782.042) and the João Paulo II Children’s Hospital (protocol #860.893).

Peripheral blood mononuclear cells (PBMCs) from buffy blood packs of 5 healthy donors in Australia were obtained from the Australian Red Blood Cross Service (authorized by Material Supply Agreement with The University of Melbourne), according to the University of Melbourne Ethics Committee approval (protocol #1239046.3). PBMCs were isolated using Ficoll-Paque, as previously described (14), stored in liquid nitrogen and thawed out in RPMI 10% of FCS one day before their use in activation assays.

### *Ex vivo* MAIT profiling by flow cytometry

Whole blood samples collected in EDTA-containing tubes were stained with anti-CD3-PerCP (clone: SP34-2), anti-TCRVα7.2/(TRAV1-2)-FITC (clone: 3C10), anti-CD161-BV605 (clone: DX12), anti-CD8-AF700 (clone: RPA-T8), and anti-CD69-APC-Cy7 (clone: FN50) for 20 minutes at room temperature (RT). After staining, the erythrocytes were lysed for 10 minutes using FACS lysing solution (BD Biosciences, San Jose, CA, USA). Then, cells were washed twice and resuspended in PBS. A minimum of 100,000 lymphocytes was acquired using a BD LSR Fortessa Flow Cytometer (BD Biosciences, San Jose, CA, USA) and the FACS DIVA software (BD Biosciences, San Jose, CA, USA). Data were analyzed using FlowJo software (version 9.3.2, TreeStar, San Diego, CA, USA).

### Leishmania culture and labelling

*L. infantum* (MHOM/BR/1974/PP75) promastigotes were grown at 26°C in a biphasic medium consisting of rabbit blood agar (Novy-McNeal-Nicolle medium) and Liver Infusion Triptose medium (LIT) supplemented with 10% of heat-inactivated fetal calf serum (FCS). *L. donovani* (a human isolate from India) promastigotes were grown in RPMI 1640 medium (Sigma, MO, USA) supplemented with 10% of FCS at 27°C and used in the early stationary phase. Promastigotes were stained with CellTracker Orange CMRA (C34551; Thermo Fisher), as previously described (65).

### *In vitro* Leishmania infection of whole blood samples

Heparinized whole blood samples were centrifuged at 1,200 × g for 10 minutes at RT for plasma removal. Cells were resuspended in RPMI 1640 medium (Sigma, MO, USA) supplemented with 10% FCS at a final concentration of 1×10^7^ cells/mL. Cell suspensions were incubated in the presence or absence of MR1 blocking (10µg/mL) antibody (clone: 26.5) prior to the addition of live promastigotes of *Leishmania infantum* at a 1:2 ratio (parasite:cells) to the cultures. After 60 minutes of incubation in the presence of parasites, 10μg/mL of Brefeldin A (Sigma, MO, USA) was added, and the samples were incubated for 4 hours at 37°C and 5% CO_2_. Cultures without MR1 blocking antibody and parasites were used as controls. Following incubation, the samples were treated with 20mmol/L of EDTA (Sigma, MO, USA) for 15 minutes at RT. Then, the cells were stained with anti-CD3-PerCP (clone: SP34-2), FITC/anti-TCRVα7.2/TRAV1-2 (clone: 3C10), BV605/anti-CD161 (clone: DX12), AF700/anti-CD8 (clone: RPA-T8), and APC-Cy7/anti-CD69 (clone: FN50) monoclonal antibodies. After 30 minutes of incubation at RT, erythrocytes were lysed for 10 minutes with FACS lysing solution (BD Biosciences, San Jose, CA, USA), and the samples were permeabilized for intracellular staining. For this, cells were incubated with PBS-P (PBS containing 0.5% BSA, 0.5% saponin and 0.1% sodium azide) for 10 minutes at RT. Following permeabilization, samples were stained with PE/anti-TNF (clone: mAb11), PE/anti-IFN-γ (clone: 4S.B3), PE/anti-IL-17A (clone: SCPL1362), and PE/anti-IL-10 (clone: JES3-9D7) monoclonal antibodies. Samples were washed with PBS-P and PBS-W (PBS containing 0.5% BSA and 0.1% sodium azide) and resuspended in PBS. A minimum of 100,000 lymphocytes were acquired using a BD LSR Fortessa Flow Cytometer and FACS DIVA software (BD Biosciences, San Jose, CA, USA). The data were analyzed by FlowJo (version 10.1, TreeStar Inc. Ashland, OR, USA).

### Assessment of cytokines upon short-term Leishmania stimuli

After whole blood cell culture under different conditions (i.e., control, *L. infantum*-stimulated and cultures with MR1 blocking antibody), the levels of TNF, IFN-γ, IL-17A, IL-12, IL-15, and the regulatory cytokine IL-10 were quantified in the cell culture supernatants were measured by high-throughput microbeads array (Bio-Plex Pro™ Human Cytokine multiplex Assay, Bio-Rad Laboratories, Hercules, CA, USA), following the manufacturer’s instructions. The results were expressed in pg/mL according to standard curves for each immune mediator using a fifth parameter logistic fit analysis. Luminex analyses were run using the Bio-plex 200 System (Bio Rad Laboratories, CA, USA) at the Flow Cytometry core facility at FIOCRUZ-MINAS.

### *In vitro* Leishmania infection of THP-1 cell lines

THP-1 cells (ATCC^®^ TIB-202™) along with THP-1 cells over-expressing MR1 (THP-1.MR1) and THP-1 cells deficient in MR1 (THP-1.MR1K0) (20) were used as antigen-presenting cells (APC) for *in vitro* activation experiments. Cell lines were differentiated in 96-well plates (50,000/well) in RPMI 1640 medium (Sigma, MO, USA) supplemented with 10% FCS and phorbol myristate acetate (PMA - Sigma, MO, USA) at 50ng/mL for 2 days at 37°C and 5% CO_2_. Differentiated THP-1 cell lines were washed three times with RPMI 1640 medium (Sigma, MO, USA) supplemented with 10% FCS and infected with *L. donovani* using 10:1 multiplicities of infection (MOI) for 4 hours at 37°C and 5% CO_2_. After infection, cells were washed three times with RPMI 1640 medium (Sigma, MO, USA) with 10% FCS and incubated overnight at 37°C, 5% CO_2_. Cells were then washed three times with RPMI 1640 medium (Sigma, MO, USA) with 10% FCS and PBMCs were added to the cultures at a 10:1 ratio (PBMC : APC). Synthetic 5-OP-RU at a final concentration of 1nM was included as a positive control and prepared as previously described (73). Samples were incubated at 37°C and 5% CO_2_ for 1 hour prior to adding Brefeldin A (Sigma, MO, USA) (10μg/mL) and incubated for another 5 hours at 37°C and 5% CO_2_. The cells were washed with PBS and stained for 20 minutes at RT using Zombie Yellow Fixable Viability Kit (Biolegend), anti- PE-CF594/CD3 (clone UCHT1), BV785/anti-TRAV1-2 (clone 3C10), PEVio770/anti-CD161 (REA631) monoclonal antibodies, and human MR1–5-OP-RU tetramer, prepared in house as previously described (14, 74). Cells were then fixed with 1% Paraformaldehyde (PFA – Sigma, MO, USA) in PBS for 20 minutes at RT, washed twice with PBS and stained with APC/anti-TNF (clone: mAb11) monoclonal antibody in PBS supplemented with 0.3% Saponin overnight at 4°C. Cells were washed twice with PBS, and a minimum of 150,000 cells per sample were acquired using an BD LSR Fortessa Flow Cytometer and FACS DIVA software (BD Biosciences, San Jose, CA, USA). Data were analyzed with FlowJo (version 10.1, TreeStar Inc. Ashland, OR, USA).

### Statistical analysis

Statistical analyses were performed using GraphPad Prism software (San Diego, USA, version 5.03). The non-parametric distribution of the data was confirmed by the Shapiro-Wilk test. The non-parametric Mann-Whitney test was used for comparative analyses between two groups. The Spearman test was employed for correlation analysis. Differences were considered statistically significant when the p-value was lower than 0.05. Categorical data analysis was performed to compare the expression of surface markers and the cytokine production by MAIT cell subsets, CD8^-^ and CD8^+^ non-MAIT T-cells, using the global median of each biomarker as the cut-off to categorize low and high results. Heatmap analysis was employed to compile the frequency of subjects with high expression of markers (above the global median).

## Data availability statement

The raw data supporting the conclusions of this article will be made available by the authors, without undue reservation.

## Ethics statement

The studies involving human participants were reviewed and approved by Rene Rachou Institute - IRR/FIOCRUZ protocol #782.042. Written informed consent to participate in this study was provided by the participants’ legal guardian/next of kin.

## Author contributions

VP-M and JC-d-R conceived the study and acquired funding. JC-d-R, ML, ÁLR, LB, VS, TP, ZC, and ES performed experiments in Brazil and Australia. MP-X, MSA, OM-F, AT-C, VP-M, and JC-d-R performed data analysis. ALC, MAM, FC, MB, and MC performed clinical sample acquisition and patient’s treatment and follow-up. MC, SE, MM, MT, and JM provided reagents, acquired additional funding and provided critical scientific advisory. VP, JC-d-R, and ML prepared figures and the first draft of manuscript. SE revised and modified the first drafts of manuscript. All authors revised and approved the final versions of the manuscript.

## Funding

JC-d-R received financial support from the Conselho Nacional de Desenvolvimento Científico e Tecnológico –MCTI/CNPq/2014 - Grant# 458134/2014-7) and a fellowship from the Brazilian National Postdoctoral Fellowship Program (PNPD/CAPES). VP-M received financial support from the Fundação de Amparo à Pesquisa do Estado de Minas Gerais (FAPEMIG – Grant# APQ-01754-14 and PPM-00497-16). This study was financed in parts by the Coordenação de Aperfeiçoamento de Pessoal de–Nível Superior –Brasil (CAPES) - Finance Code 001. SE was supported by a fellowship from the Australian Research Council (ARC) (ARC DECRA DE170100407) and a fellowship and project grant from the Australian National Health and Medical Research Council (NHMRC fellowship APP1196881 and project grant GNT1157388). JC-d-R (Pq2), OM-F (Pq1B), AT-C (Pq1D), MC (Pq1D), and MSA (Pq2) thank CNPq for their Senior Research Productivity Fellowships. MT received financial support from the National Institutes of Health (R01-AI070258).

## Acknowledgments

We thank Fernanda Vianni and Daniela Caldas Teixeira for medical assistance. We acknowledge the Melbourne Cytometry Platform (Doherty Institute node) for providing flow cytometry services.

## Conflict of interest

JM, ZC, and SE are inventor of patents describing MR1 antigens and MR1 tetramers.

The remaining authors declare that the research was conducted in the absence of any commercial or financial relationships that could be construed as a potential conflict of interest.

## Publisher’s note

All claims expressed in this article are solely those of the authors and do not necessarily represent those of their affiliated organizations, or those of the publisher, the editors and the reviewers. Any product that may be evaluated in this article, or claim that may be made by its manufacturer, is not guaranteed or endorsed by the publisher.
