# Supplementary figures and images for: The role of mucosal-associated invariant T cells in visceral leishmaniasis
Source: Front Immunol. 2022 Sep 15;13:926446. doi: 10.3389/fimmu.2022.926446 (PMC9521739; doi:10.3389/fimmu.2022.926446)

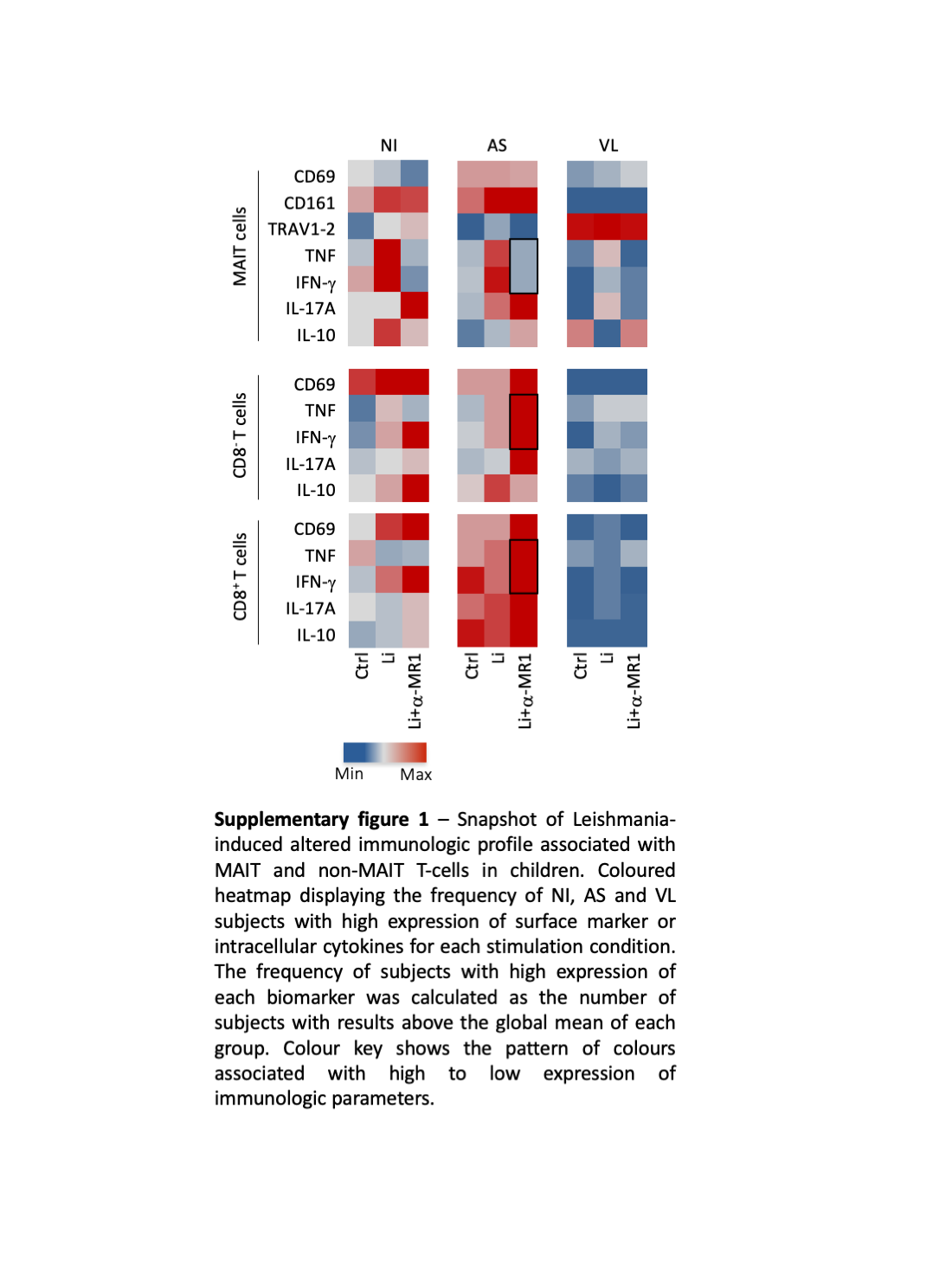

Supplement: Supplementary file 1 [file Image_1.tiff]
